# Missed Congenital Pyloric Atresia with Gastric Perforation in a Neonate

**Published:** 2012-04-01

**Authors:** Yousuf Aziz Khan, Naima Zamir

**Affiliations:** Department of Pediatric Surgery, National Institute of Child Health Karachi, Pakistan

**Dear Sir,**

Congenital pyloric atresia is a rare anomaly accounting for less than 1% of upper GI atresias. It may occur in isolation or in association with other congenital anomalies, epidermolysis bullosa being the most frequent. It presents with upper abdominal distension, non-bilious vomiting or rarely with complications as aspiration pneumonia, electrolyte imbalance or gastric perforation. Though iatrogenic gastric perforations secondary to aggressive resuscitation with bag-mask ventilation and nasogastric intubation are the most frequent in newborns, neonatal gastric perforation due to congenital outlet obstruction have rarely been reported [1-5]. The clinical course of a newborn is worth sharing who presented with gastric perforation and had an underlying pyloric atresia that was missed.

A two days old pre-term (weight 1.6 kg) male newborn was referred to our institute from a remote area. According to his parents, he was delivered by caesarean section and admitted in NICU for respiratory distress. There was no history of aggressive resuscitation and/or mechanical ventilation. Ante-natal ultrasound had shown polyhydramnios. He had passed meconium in small amount few hours after birth and on the 2nd of life, developed gross abdominal distension.

On arrival, the baby was sick, lethargic and hypothermic with poor peripheral perfusion. He was in respiratory distress, chest was clear and had oxygen saturation of 74% at room air. Abdomen was grossly distended, and tense. Anal orifice was normal. X-ray abdomen revealed massive pneumoperitoneum. As a resuscitative measure, 16G I.V cannula was inserted in the epigastrium to relieve tension pneumoperitoneum, followed by tube laparostomy which drained only small amount of hemorrhagic fluid. After optimization of his general condition, laparotomy was performed. A 3×3 cm perforation was found at the fundus of stomach; distally small bowel was collapsed and peritoneal cavity was clean. Gastrorrhaphy was performed and abdomen was closed at that stage. Post operatively, he developed septicemia but recovered ultimately. NG feed was allowed on the 8th post operative day which was not tolerated. There was upper abdominal fullness with continuous non-bilious NG aspirate and X-ray abdomen showed paucity of gases beyond stomach. Upper GI contrast study was done, which revealed contrast filled, distended stomach and failure of passage of contrast beyond, suggestive of gastric outlet obstruction (Fig.1). He was re-explored and type I pyloric atresia was found which was excised and Heineke Mikulicz pyloroplasty was performed. Post operative course was then uneventful. He was allowed oral feed on 6th post op day (of 2nd surgery), which was tolerated well. It was gradually increased and he was discharged to home.

**Figure:**
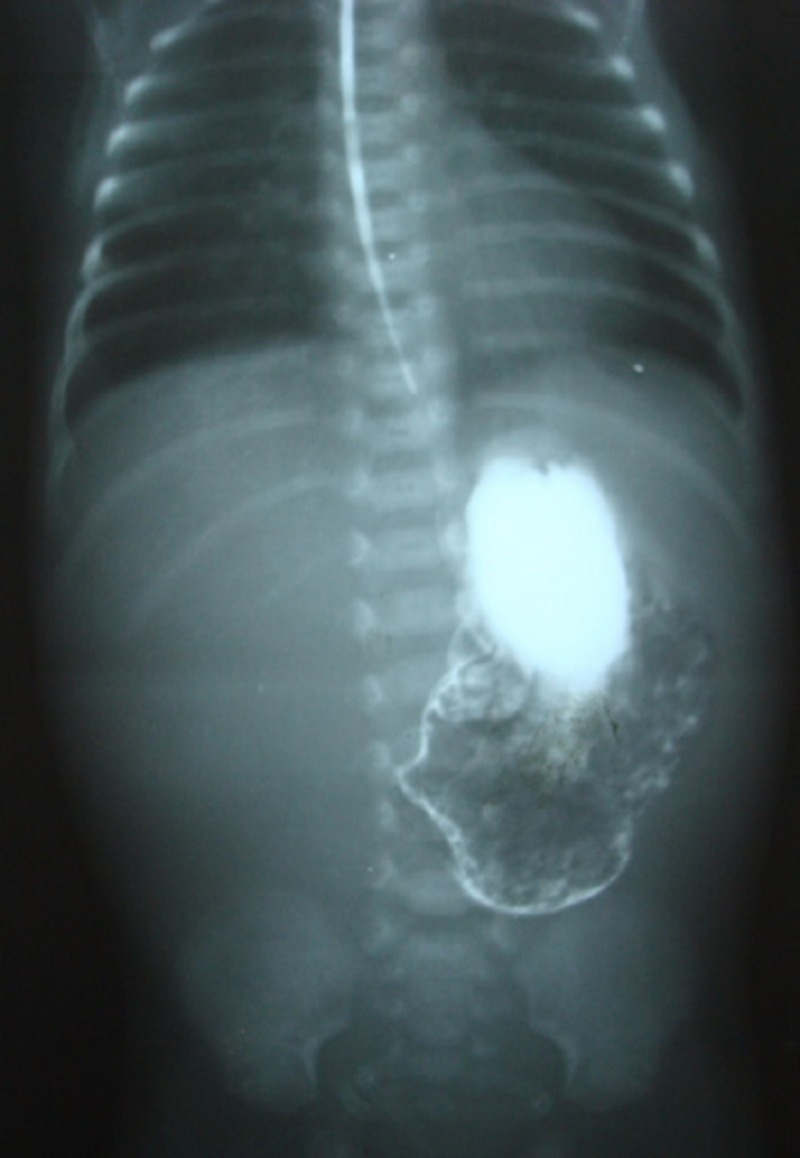
Figure 1: Upper GI barium study of the baby showing dilated stomach with failure of the passage of contrast beyond.

Though uncommon, congenital causes of gastric outlet obstruction such as a type I pyloric atresia must not be forgotten and missed while operating upon a newborn with gastric perforation. Had it been in mind, the baby wouldn’t have undergone re-exploration. Distending the stomach with normal saline after repair of perforation would be sufficient to confirm distal patency. An ante-natal ultrasound shouldn’t be underestimated as it may give clue to the diagnosis.

## Footnotes

**Source of Support:** Nil

**Conflict of Interest:** None declared

